# C-reactive protein, glucose and iron concentrations are significantly altered in dogs undergoing open ovariohysterectomy or ovariectomy

**DOI:** 10.1186/s13028-018-0384-6

**Published:** 2018-05-30

**Authors:** Elena Regine Moldal, Mads Jens Kjelgaard-Hansen, Marijke Elisabeth Peeters, Ane Nødtvedt, Jolle Kirpensteijn

**Affiliations:** 1 Department of Companion Animal Clinical Sciences, Faculty of Veterinary Medicine and Biosciences, Norwegian University of Life Sciences, Oslo, Norway; 2 Department of Veterinary Clinical Sciences, Faculty of Health and Medical Sciences, University of Copenhagen, Copenhagen, Denmark; 3 Department of Clinical Sciences of Companion Animals, Faculty of Veterinary Medicine, University of Utrecht, Utrecht, The Netherlands; 4 Department of Production Animal Clinical Sciences, Faculty of Veterinary Medicine and Biosciences, Norwegian University of Life Sciences, Oslo, Norway; 5 Hill’s Pet Nutrition Inc, Topeka, KS USA

**Keywords:** C-reactive protein, Glucose, Iron, Ovariectomy, Ovariohysterectomy, Surgery, Surgical stress response

## Abstract

**Background:**

There are relatively few studies about the canine surgical stress response, a sequence of events orchestrated by the body in response to a surgical trauma which is sometimes, as shown in human surgery, deleterious to the patient. There is a need to identify objective markers to quantify this response in order to estimate tissue trauma and use the markers as potential early indicators of surgical complications. The study objective was to investigate the surgical stress response, measured by C-reactive protein (CRP), glucose and iron serum concentrations, to gonadectomy in female dogs, and to compare the response to ovariohysterectomy (OHE) with the response to ovariectomy (OVE). A randomized clinical trial was performed on a sample of 42 female dogs, which were divided into two groups: one group underwent OHE, the other OVE.

**Results:**

Blood samples were collected immediately before surgery (T0), and at 1 (T1), 6 (T6), and 24 (T24) h after surgery, and serum frozen and stored at − 80 °C for later analysis. Upon thawing, the serum samples were subjected to measurement of CRP, glucose and iron concentration. Seventeen dogs in the OHE group and 19 dogs in the OVE group were included in the statistical analysis. There was a significant increase in glucose concentration at all time points compared with T0, and an increase of CRP at T6 and T24. Iron concentration was significantly decreased at T6 and T24. Differences between the two groups could not be detected for any of the three variables.

**Conclusions:**

The study showed that both OHE and OVE induce a moderate surgical stress response in female dogs, measured by CRP, glucose and iron. A difference between the surgical techniques could not be detected for any of the variables, and hence; with regards to the parameters studied recommendations of one procedure over the other cannot be made and preferred technique remains the surgeon’s choice.

## Background

The stress response to surgery involves an array of physiological events in the body, including endocrinological, immunological, and hematological alterations leading to a catabolic state [1, 2]. Even though these functions are beneficial in the acute survival situation, this response may in fact have negative effects on homeostasis and tissue healing [3].

The surgical stress response is believed to be proportional with the degree of tissue injury caused by the procedure [4, 5]. It is therefore important to choose surgical procedures that minimize the negative impact of surgery on the body. Complications after elective surgery in dogs and cats are not uncommon and have been reported to include hemorrhage, surgical site inflammation or infection, and increased attention to the surgical site [6, 7]. Female dogs are commonly neutered, most often by open ovariohysterectomy (OHE) or ovariectomy (OVE). Several authors argue that OVE should be the preferred method because of the belief that it is faster, safer, less invasive, and associated with fewer postoperative complications [8–10]. Open OHE in dogs has previously been shown to induce a significant, but short-lived neuroendocrine stress response [11]. Two previous studies by the authors comparing OVE and OHE failed to show differences between the two methods with regards to pain scores, time expenditure, and wound characteristics, as well as difference in the hemostatic stress response to surgery [12, 13]. However, one recent study identified significant differences in postoperative C-reactive protein (CRP) concentrations in three groups of dogs subjected to vasectomy, open OHE, or laparoscopic OHE [14]. CRP is an acute phase protein and a sensitive marker of inflammation [15–17], and can be used to quantify the inflammatory response to different surgical procedures in dogs [14]. Glucose is another biomarker commonly used to measure the stress response to surgery. A study comparing dogs subjected to open OHE with dogs subjected to the laparoscopic counterpart identified prolonged increases in glucose concentration in the open OHE group during the postoperative period [18]. Glucose is also an independent risk factor for postoperative wound infections in humans [19]. Hypoferremia is commonly seen after surgically induced inflammation in humans and is related to the extent of surgery [20]; however, information about iron concentration after surgery in dogs is scarce.

The aims of this study were to measure CRP, iron and glucose serum concentration as markers of the surgical stress response in dogs, and to test whether they differed between two commonly applied methods for surgical neutering, of which one—OVE—has been claimed to be less traumatic by some authors [8–10]. The hypothesis tested was: Surgery will cause significant increases of serum CRP and glucose and a decrease in serum iron-concentrations postoperatively, but to a lesser degree in the OVE compared to the OHE group.

## Methods

The study was approved by the Ethics and Research Committee of the Department of Clinical Sciences of Companion Animals, Faculty of Veterinary Medicine, University of Utrecht (DCSCA), the Netherlands. It was performed as a prospective randomized clinical trial at the DCSCA between June 2006 and June 2007. Serum was stored at − 80 °C for a maximum of 4 years, and later transported to the University of Copenhagen on dry ice before analysis at the Central Laboratory, Department of Veterinary Clinical Sciences, University of Copenhagen, Denmark, in November 2010. The laboratory analysis was performed double blind in one analytical run, in random order, and unblinding did not take place until after statistical analysis of the data. Only the surgeon (MEP) knew what procedure was performed.

### Study population

A total of 42 client-owned healthy intact bitches admitted to the DCSCA for elective neutering were prospectively entered into the study. Of these, 12 bitches were mongrels and 30 were pure-bred. Oral consent was obtained from the owners before the dogs underwent a thorough clinical examination to ensure that they were healthy. Only dogs assigned to ASA category 1 (normal, healthy animals) [21] were eligible for participation in the study, and all dogs went through their last estrus at least 6 weeks prior to presentation. Each dog was given a body condition score (BCS) at admission, with a score of 1 being emaciated and 5 being obese. The dogs were numbered consecutively at admission. Dogs were block randomized into one of two treatment groups, OVE or OHE, after induction of anesthesia [12].

### Anesthesia, surgery, and analgesia

An intravenous (IV) catheter was inserted in the cephalic vein. The dogs were given a premedication of 1 mg/m^2^ medetomidine intravenously (Domitor, Pfizer Animal Health, USA, 1 mg/mL) and 4 mg/kg carprofen IV (Rimadyl, Pfizer Animal Health, USA, 50 mg/mL) and anesthesia was induced with 1–2 mg/kg propofol IV (PropoVet, Abbott Laboratories, UK, 10 mg/mL) to effect. The dogs were then intubated and anesthesia was maintained with isoflurane (Isoflo, Abbott Laboratories, UK) in oxygen and air. Intermittent positive pressure ventilation (IPPV) was applied to ensure normocapnea and the volume was regulated to keep end-tidal CO_2_ at normal levels (4.5–5 kPa). All dogs were given 10 mL/kg/h Ringer’s lactate IV (Stereofundin, Iso; B, Germany) at maintenance rate throughout the course of anesthesia and surgery. Intraoperative monitoring consisted of electrocardiogram (ECG), capnography, body temperature, and oxygen and vapor concentrations. In surgeries that lasted for more than one h, an additional dose corresponding to half of the original administered dose of medetomidine was administered IV. After surgery this was antagonized with 2.5 µg/m^2^ atipamezole intramuscularly (IM) (Antisedan, Pfizer Animal Health, USA, 5 mg/mL) [12].

All surgeries were performed by one experienced ECVS Diplomate (MEP) with the help of an assistant, using a standardized surgical protocol for both procedures. Both OVE and OHE were carried out as open surgical procedures. The OVE dogs had their ovaries removed through a smaller incision than the OHE dogs, which additionally had their uterus removed [12].

All dogs were hospitalized for 24–32 h postoperatively. 10 µg/kg buprenorphine (Buprecare, Animalcare Ltd, UK, 0.3 mg/mL) was administered IV approximately 40 m before injecting atipamezole and then given subcutaneously (SC) every 6 h during the next 24 h. The rescue analgesia protocol consisted of administration of a higher dose of buprenorphine 20 µg/kg SC to animals showing pain scores > 15 on a modified version of the Short Form (SF) of the Glasgow Composite Measure Pain Scale [22]. Treatment at home consisted of 2 mg/kg carprofen orally every 12 h for an additional 2 days after discharge [12].

### Blood sampling

Immediately after anesthetic induction an IV jugular catheter was inserted and secured in place. Just before the skin incision (T0) and just before closure of the abdominal incision (T1), and also at 6 h after T0 (T6), blood samples were collected from this catheter after discarding the first 5 mL of blood. The jugular catheter was then removed, and the 24-h blood sample (T24) was taken by direct venipuncture of the contralateral jugular vein. For all samples, a total of 11 mL blood was collected in one serum tube and two 3.2% citrate tubes, in that order. For T0, 10 additional mL blood was collected in heparin and EDTA for biochemistry and hematology, to confirm the animal’s health before enrolment in the project. The following variables were analyzed: BUN (blood urea nitrogen), serum creatinine, alkaline phosphatase, bile acids, total plasma calcium, phosphorus, sodium, potassium, hematocrit, total leucocytes, and platelets.

All serum tubes were left in room temperature and centrifuged after 1 h at 4 °C at 1006*g* for 10 min before the serum was separated and placed directly in a − 80 °C freezer for later analysis at the Department of Veterinary Clinical Sciences.

### Other

Hemostasis parameters and other variables including blood loss, surgical time, surgical wound characteristics, pain scores, and wound assessment scores were recorded and published in other studies [12, 13].

### CRP

CRP levels were analyzed using a turbidimetric immunoassay (High Linearity CRP, Randox Laboratories Ltd., Crumlin, UK) performed on Advia 1800 Chemistry System (Siemens, Germany). Independently purified canine CRP was applied as calibrator (cat#8101, Life Diagnostics, West Chester, PA, USA) and control (TP-810CON, Tridelta, Kildare, Ireland). For complete assay performance, please see validation conducted by the laboratory performing the measurements [23, 24]. Automated reflex dilution was applied when measurement exceeded linear range, resulting in effective working range up to 600 mg/L. No prozone effect were observed up to 900 mg/L.

### Glucose

Glucose was measured with the reagent Glucose Hexokinase/Glucose oxidase, including assay calibrator provided by manufacturer (Siemens, Germany) performed on the Advia 1800 Chemistry System. Imprecision was below 2%.

### Iron

Iron was measured by using the reagent Iron RGT KT D/S, including calibrator provided by manufacturer (Siemens, Germany) on the Advia 1800 Chemistry System. Imprecision was below 2%.

### Statistical analysis

Two dogs were excluded from the study, one because it was under treatment with phenobarbital for epilepsy, the other because of unexpected complications during surgery which lengthened the procedure but were not associated with the procedure per se. Also, because four serum samples were stored in a different freezer for a period of time, one sample from the OVE group and three samples from the OHE group were discarded. Thus, results from 36 dogs, 17 in the OHE group and 19 in the OVE group, were included in the statistical analysis. All statistical analyses were performed using the statistical software package Stata version 11 (Statacorp, College Station, USA). Three separate regression analyses were performed; one for each of the outcome variables CRP, glucose and serum iron concentrations. The explanatory variables were treatment group (OVE or OHE) and time [0 (= baseline), 1, 6, 24 h] in all models. Variables were initially evaluated for correlations between time points. Observations within each dog through time were not independent of each other. Therefore linear mixed regression models, including random effects for dog, were applied to detect differences between the treatment groups and between time points for each of the outcome variables. The overall effect of the categorical variable time was tested using likelihood ratio (LR) tests. The level of statistical significance was set to P < 0.05. The assumption of normally distributed residuals was assessed using normal quantile plots at the dog level.

## Results

The mean age of participating dogs in the sample was 3.4 years, range 6 months to 10 years, and the mean weight 25 kg, range 12–36 kg. The groups did not differ with regards to age, body weight, body condition score, and surgical time [12]. Preoperative biochemical and hematological profiles in the dogs were within the reference intervals of the DCSCA. None of the dogs had pain scores > 15 and thus, rescue analgesia was not indicated in any of the animals.

### CRP, glucose and iron

**Fig. 1:**
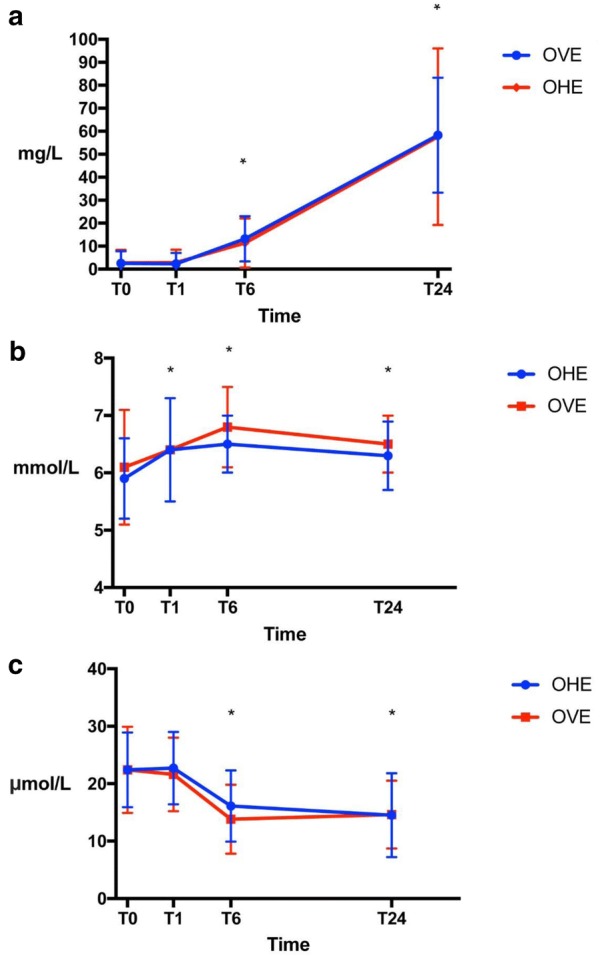
Mean CRP (**a**), glucose (**b**), and iron (**c**) concentrations for the OHE and OVE group at each time point T0, T1, T6 and T24. There was no difference between groups for any of the parameters but the statistically significant changes from T0 are marked with asterisks

Mean and standard deviation for CRP, glucose and iron serum concentrations by time and group are presented in Table 1. The baseline (T0) values did not differ significantly between the groups for any of the three variables. Based on the observed correlations, an exchangeable correlation structure between time points was assumed for glucose and CRP, and a first-order autoregressive for iron concentration. The reported effects of treatment group and time are based on output from the three regression models for CRP, glucose and iron (Model output available from the first author by request). CRP (Fig. 1a) was increased at T6 and T24 (P < 0.001) for both groups. There was no significant difference in CRP between groups (P = 0.92). The glucose concentration (Fig. 1b) was higher than baseline (T0) at all time points (LR test of group; P = 0.004), but no difference between groups was detected (P = 0.27). Iron concentration (Fig. 1c) was decreased at T6 and T24 compared to baseline (P < 0.001 for both), with no difference between groups (P = 0.68). Residuals were approximately normally distributed for all three models when assessed at the dog-level using normal quantile plots. The random dog-effect was highly significant for all three variables.

**Table 1:** Mean and standard deviation (SD) of CRP, glucose, and iron serum concentrations for dogs in the OHE and OVE group

Variable	Time	OHE mean	SD	OVE mean	SD	Reference interval
CRP (mg/L)	0	2.7	5.7	2.5	5.3	0.4–15.9
	1	2.9	5.7	2.3	4.8	
	6	11.4	10.7	13.2	9.8	
	24	57.6	38.4	58.3	25.0	
Glucose (mmol/L)	0	5.9	0.7	6.1	1.0	3.9–6.6
	1	6.4	0.9	6.4	0.9	
	6	6.5	0.5	6.8	0.7	
	24	6.3	0.6	6.5	0.5	
Iron (µmol/L)	0	22.4	6.5	22.4	7.5	5.4–32.2
	1	22.7	6.3	21.6	6.4	
	6	16.1	6.2	13.8	6.0	
	24	14.5	7.3	14.6	5.9

## Discussion

Both OHE and OVE induced significant postoperative changes in CRP, glucose and iron concentrations. The hypothesis that OVE would cause a less marked stress response could however not be supported, which corroborates the authors’ two previous studies comparing OVE and OHE [12, 13]. The detection of increased CRP after surgery is in accordance with previous human and canine studies [14–17, 25–28]. Increased glucose concentration perioperatively has also been registered in both species [11, 18, 19, 29].

Decreased iron concentration has been reported both after soft tissue and orthopedic surgery in humans [20, 30], and the magnitude of this decrease differs with surgical invasiveness [20]. Information about iron concentration in dogs after surgery is scarce, but unpublished observations by the authors indicate decreased concentrations after both skin-, abdominal- and orthopedic surgery compared with pre-operative values in dogs.

CRP is a major acute phase protein in dogs and the results expectedly indicate that a moderate inflammatory response occurs after both OVE and OHE. CRP has been shown to be a sensitive marker of inflammation and further has the ability to distinguish inflammatory states as a result of neoplasia, immune-mediated disease, surgery, and infections [14, 31–35]. It has been argued that CRP should be part of routine diagnostic testing because of its higher sensitivity than WBC [36, 37]. CRP can increase up to 95 times as a result of surgery [26], and this increase is related to the degree of tissue injury in dogs [14, 26]. Thus, CRP can be used to reflect the degree of surgical trauma [14]. In our study CRP increased approximately 20-fold from T0 to T24. There was no difference between groups. OHE has previously been shown to cause moderately elevated CRP in dogs [16, 26]; however, to a lesser degree than more invasive surgery like orthopedic surgery [26]. In a study of humans, a smaller elevation of CRP was detected after laparoscopic hysterectomy compared to the open abdominal procedure [38], and the same phenomenon has been identified in dogs [14]. The results from the current study serve to indicate that tissue trauma, as measured by CRP, is comparable for open OVE and OHE.

The glucose concentration significantly increased at T1 and T6, but slightly decreased again at T24; however, the difference from T0 to T24 was still statistically significant. There was no difference between the two groups. Blood glucose concentration is a useful measure of surgical stress in dogs [18], and has been identified as an independent risk factor for infection after surgery in humans [19, 29]. Hyperglycemia has deleterious effects on macrophage and neutrophil function [39], and this may explain why human patients suffering from diabetes mellitus are twice as likely to develop a post-operative infection compared to normoglycemic individuals [40, 41]. The pathophysiology behind postoperative hyperglycemia is partly induction of a hyperglycemic response by cortisol and growth hormone and partly insulin resistance and inhibition of insulin secretion, all induced by the neuroendocrine and metabolic stress response to surgery [5]. Glucose concentration has also been shown to have predictive value on the outcome in critically ill human patients [42]. In a study by Benson et al. [11], glucose was found to be elevated after anesthesia and surgery (OHE) in dogs. The increasing glucose concentration up to T6 corroborates a previous study on OHE in dogs [18]. In a study by Hardie et al. [43], 50% of dogs with sepsis that developed high glucose concentrations postoperatively died, whereas mortality in the group with normal glucose concentration was 14%. The difference was, however, not statistically significant (P = 0.08) [43]. The link between high glucose concentration and morbidity is not completely understood, but it has been suggested that the responsiveness of leukocytes stimulated with inflammatory mediators is inversely correlated with indices of in vivo glycemic control in humans [39]. As a minor study limitation it should be noted that time of postoperative feeding is not available for the dogs in the study. Also, because the postoperative glucose concentration was in the upper end of, and not outside, the reference interval for dogs in our study, a clinical relevance is considered unlikely. Nevertheless, it seems that OHE and OVE induce increased glucose concentration to a comparable extent.

The iron concentration decreased to a similar degree in both groups after surgery, at T6 and T24. An anemic state that resembles anemia of chronic disease commonly occurs in humans after surgery [20, 30], and can take up to 6 weeks to normalize [30]. This was previously believed to be purely due to blood loss; however, iron supplementation after orthopedic surgery has no major effect on erythropoiesis [44, 45]. Research in mice indicates that hypoferremia is mediated by interleukin 6 (IL-6) because it induces synthesis of the iron regulatory hormone hepcidin, an acute phase protein in humans [46, 47]. Transferrin, an iron binding transporter protein, is also a negative acute phase protein in dogs [31]. There are great similarities between dogs and humans in iron metabolism [48], and the mechanisms triggered postoperatively are likely to be similar as well. The iron concentration decreased to a similar extent in both groups.

It should be noted that several factors may influence the surgical stress response. Stress caused by hospitalization is commonly seen in dogs and may exacerbate the endocrine responses to surgery [18]. Care must be taken to avoid stress in surgical patients in order to minimize the catabolic events mediated by the stress response. This can in part be done with sedative and anesthetic drugs. In this study, medetomidine was used for premedication. Medetomidine has been shown to obtund the surgical stress response by preventing the catecholamine response induced by OHE [11], and could therefore have affected the glucose concentration to some degree. There is no evidence in the literature to say that medetomidine has an anti-inflammatory effect, and hence, an influence on CRP and iron concentration is considered unlikely. One could argue that the use of non-steroidal anti-inflammatory drugs (NSAIDs) such as carprofen would limit the inflammatory response to surgery; however, it is believed that NSAIDs do not directly block the production of IL-6 [49], which is proposed to be the main inducer of CRP [26, 50]. Also, it has previously been shown that CRP and iron as inflammatory markers are not affected by NSAID administration in humans [51], and neither meloxicam nor carprofen administration caused lower postoperative concentrations of CRP in a study of OHE in dogs [28]. Also, since carprofen administration would impact the two groups to a similar extent, we consider it a minor limitation to the study. The effects of stress and administration of anesthetic and analgesic drugs are also assumed to be similar for both groups, but it cannot be excluded that the drugs have masked the surgical stress response and hence masked a potential small difference between groups. A previous study has shown higher CRP concentrations after canine OHE performed by inexperienced surgeons [27]; however, since we used the same, experienced surgeon for all procedures, this is not relevant for the current study. The dogs were only followed for 24 h, and a follow-up to assess wound healing or inflammatory complications was not carried out. In humans, increased perioperative concentrations of glucose and CRP have been described as risk factors for postoperative infections [19, 52]. A study with longer follow-up of the animals with regards to complications resulting from surgery would have been of value.

The results from the current study show that open OVE and OHE provoke a moderate surgical stress response, as measured by CRP, glucose and iron concentration, of similar magnitude, likely because the two methods are too similar in surgical invasiveness to detect subtle differences. Laparascopic techniques may confer advantages over OHE and OVE in limiting inflammation and pain in the postoperative period [14, 18, 53–55].

## Conclusions

The study showed that OHE and OVE induce a surgical stress response with postoperative increases in glucose concentration and CRP, and a decrease in iron concentration. No significant difference between the OHE and OVE group could be detected with regards to the parameters measured, and a recommendation of one procedure over the other can therefore not be made based on the findings of this study.

## Abbreviations

CRP: C-reactive protein
DCSCA: The Department of Clinical Sciences of Companion Animals, Faculty of Veterinary Medicine, University of Utrecht
IL-6: interleukin 6
NSAIDs: non-steroidal anti-inflammatory drugs
OHE: ovariohysterectomy
OVE: ovariectomy
